# Calculating the most likely intron splicing orders in *S. pombe*, fruit fly, *Arabidopsis thaliana*, and humans

**DOI:** 10.1186/s12859-020-03818-6

**Published:** 2020-10-24

**Authors:** Meng Li

**Affiliations:** CAS-MPG Partner Institute for Computational Biology, Shanghai Institutes for Biological Sciences, Chinese Academy of Sciences, Shanghai, China

**Keywords:** Splicing, Intron splicing order, Most likely order, Bayesian network

## Abstract

**Background:**

Introns have been shown to be spliced in a defined order, and this order influences both alternative splicing regulation and splicing fidelity, but previous studies have only considered neighbouring introns. The detailed intron splicing order remains unknown.

**Results:**

In this work, a method was developed that can calculate the intron splicing orders of all introns in each transcript. A simulation study showed that this method can accurately calculate intron splicing orders. I further applied this method to real *S. pombe*, fruit fly, *Arabidopsis thaliana*, and human sequencing datasets and found that intron splicing orders change from gene to gene and that humans contain more not in-order spliced transcripts than *S. pombe*, fruit fly and *Arabidopsis thaliana*. In addition, I reconfirmed that the first introns in humans are spliced slower than those in *S. pombe*, fruit fly, and *Arabidopsis thaliana* genome-widely. Both the calculated most likely orders and the method developed here are available on the web.

**Conclusions:**

A novel computational method was developed to calculate the intron splicing orders and applied the method to real sequencing datasets. I obtained intron splicing orders for hundreds or thousands of genes in four organisms. I found humans contain more number of not in-order spliced transcripts.

## Background

Splicing has been shown to be an integrated process coupled with transcription [1], and the co-transcriptional nature of splicing has been shown in various ways, such as via the sawtooth pattern of RNA-seq [2], real-time imaging [3], nuclear fraction RNA-seq [4], and electron imaging for direct visualization of co-transcription [5]. These results showed that most introns in higher organisms are co-transcriptionally spliced.

One natural line of thought is that since splicing is coupled with transcription, the splicing order may also be consistent with the transcriptional direction [6]. However, several recent studies have shown that this is not the case; these studies used long-read sequencing or bulk short-read sequencing to find that splicing is a co-transcriptional process but is not always consistent with the direction of transcription in *S. pombe*, fruit fly and humans [6–8]. In addition, intron splicing order has been shown to influence alternative splicing in *COL5A1 *[9], and the importance of intron splicing order has also been indicated by two recent studies that proved that intron splicing order can affect splicing fidelity [10, 11].

The current methods focused on neighbouring intron splicing order pairs and cannot analyse all the introns in a transcript. A computational method was developed here to accurately calculate the intron splicing order in each transcript. The method requires sequencing reads from ribo-minus depleted short-read mate-pair sequencing or ribo-minus depleted long-read sequencing. A simulation study showed that the method developed here can accurately calculate intron splicing orders. While using published long-read sequencing and short-read sequencing datasets, I calculated intron splicing orders for hundreds or thousands of genes in *S. pombe*, fruit fly, *Arabidopsis thaliana*, and humans. The results suggest that although splicing is a co-transcriptional process, the splicing order varies from gene to gene. I found that humans contain more not in-order spliced transcripts than *S. pombe*, fruit fly, and *Arabidopsis thaliana*. In addition, I confirmed that the first introns tend to be spliced slower in humans than in *S. pombe*, fruit fly, and *Arabidopsis thaliana* genome widely. The results of this work are available in [12].

## Results

**Fig. 1:**
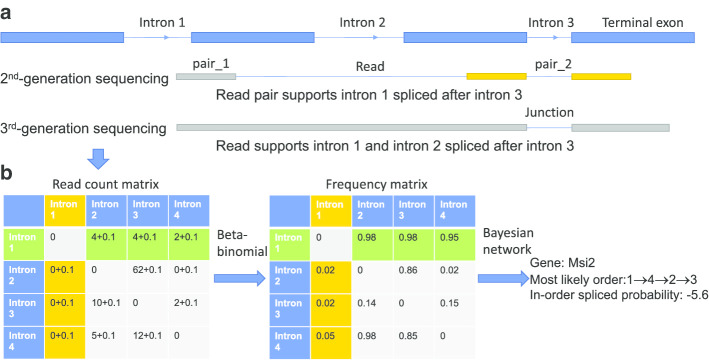
Methodology overview. **a** Sequencing methods that can detect intron splicing order pairs used by previous studies. From top to bottom: gene structure; short-read pair; long-read sequencing read. **b** The number in the adjacent matrix is the read count that supports each intron splicing order pair; the row was spliced before the column; the value within [i, j] records the read count of intron i spliced before intron j. The + 0.1 in the matrix is the pseudo read count. The right-most part of **b** represents the calculated most likely intron splicing order for this transcript, and the log relative likelihood indicates the probability that this transcript is spliced in an order that is consistent with transcription, i.e., 1 → 2 → 3 → 4

Both short-read and long-read sequencing were used to obtain information on intron splicing orders as previously stated, and the methods are outlined in Fig. 1a, which shows how the short-read pair and long-read sequencing data indicate that intron 3 is spliced before intron 1 (another example in Additional file 1). If one can calculate the intron splicing orders for each pair of introns in a transcript, then one can deduce an overall most likely intron splicing order from these intron splicing order pairs. Thus, first, these pairs can be used to fill a read count adjacent matrix, and then, the splicing frequency within each intron splicing order pairs can be obtained. Finally, the most likely order of introns can be calculated from the frequency matrix (Fig. 1b and Additional file 1). The most likely order is 1 → 4 → 2 → 3 in this example. The read counts that support intron 1 is spliced before other introns are larger than other those for introns spliced before intron 1 in Fig. 1b (read count values in row 1 larger than values in column 1), which is consistent with the most likely order calculated.

### A simulation study showed that this method worked correctly

**Fig. 2:**
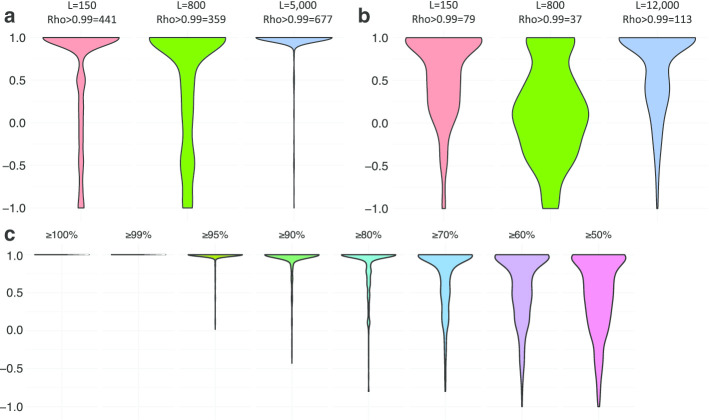
Simulation result. **a** Evaluation of the method using simulated datasets based on the *S. pombe* transcriptome; only transcripts that contain at least 3 introns are considered (n = 709). (Left violin) Evaluation of the method using simulated short-read pair-end sequencing (read length = 150). (Middle violin) Evaluation of the method using simulated long-read sequencing (read length = ~ 800). (Right violin) Evaluation of the method using simulated super long-read sequencing (read length = ~ 5000). **b** Simulation result for humans; a total of 226 multi-introns (intron number $\ge 3$) containing transcripts are used. (Left violin) Mate-pair sequencing, the fragments size = ~ 15,000 and the read length = 150. (Middle violin) Long-read sequencing, read length = ~ 800 bp. (Right violin) Super long-read sequencing, read length = ~ 12,000. **c** Simulating the intron splicing order read count matrix 1000 times by giving random orders and then randomly erasing some values in the intron splicing order read count matrix. The percentage of intron splicing order pairs retained is labelled above each violin plot

To evaluate the framework developed here, the nascent RNA sequencing reads were simulated from *S. pombe* given random intron splicing orders for each transcript, and then, the most likely orders were calculated from the simulation data using the method developed here. I performed a Spearman correlation analysis between the calculated orders and given orders to test if the intron splicing orders were correctly calculated (to test if Spearman Rho = 1). I evaluated the pipeline using both long-read sequencing and short-read sequencing. The results showed that the calculated intron splicing orders in most transcripts were the same as the given random intron splicing orders (Fig. 2a). Thus, this result proved that the methods developed here can accurately calculate intron splicing orders using short-read and long-read sequencing. The accuracy increases when the sequencing read length becomes longer, as shown in Fig. 2a, and approximately 95% of the calculated orders were the same as the input random orders when the read length was ~ 5000 bp (Fig. 2a). The same simulation was performed for human chr21 but with a much longer simulated fragment size (Fig. 2b). This simulation was also performed with different sequencing depths to find appropriate sequencing depth (Additional file 1). The reason for the relatively poor simulation result based on humans was that reads could not capture all the intron splicing order pairs in each human transcript. This also indicates that a read length of ~ 800 cannot properly capture all the intron splicing orders in humans (Fig. 2b). When the intron splicing order read count matrix was properly filled, the model performed very well, as will be shown (Fig. 2c).

In a real dataset, not each pair of intron splicing orders will be sequenced, and this problem is especially important for higher organisms, such as humans, who usually harbour very long introns. To evaluate how many intron splicing order pairs are necessary to accurately calculate the most likely orders, intron splicing order read count matrixes were generated by giving random orders and erasing some intron splicing order pairs to simulate a real dataset with missing values. Then, the calculated most likely orders were correlated with randomly given orders to test consistency. The percentage of intron splicing order pairs retained = (# of detected intron splicing order pairs) divided by (total number of intron splicing order pairs). The calculated most likely orders are relatively accurate when this measure is > 90% and very accurate when this measure is > 95% (Fig. 2c). Thus, 95% was used as a threshold for the downstream real data analysis.

### Splicing order change from gene to gene in *S. pombe*, fruit fly, *Arabidopsis thaliana* and humans

**Fig. 3:**
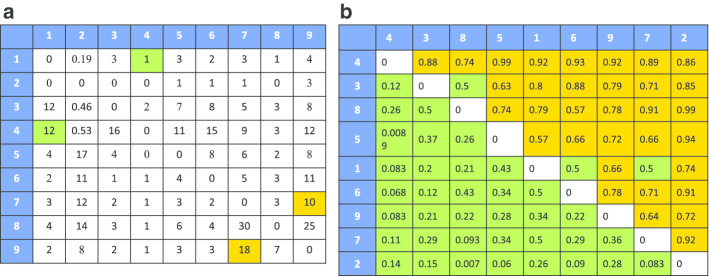
Intron splicing order of *hnRNP A1*. **a** The intron splicing order read count adjacent matrix of *hnRNP A1*. The intron index number is labelled in the first row and first column. **b** Reordering of the read count frequency matrix by most likely order; values $\le$ 0.5 and > 0.5 are coloured differently. The values in the upper diagonal are larger than the values in the lower diagonal, which supports the most likely order calculated. For example, values in row 1 (corresponding to intron 4) $\approx$ 0.9 and values in column 1 $\approx$ 0 suggest that intron 4 was spliced before other introns

I used long-read sequencing datasets and paired-end short-read sequencing datasets to perform real data analysis. For each transcript, a read count adjacent matrix and graph can be built, and the example of *hnRNP A1* is shown below (Fig. 3a). Almost every cell in the matrix has read count support, which suggests that the splicing of *hnRNP A1* frequently occurs out of order for some intron pairs, e.g., intron 7 spliced before intron 9 has 10 reads in support, while intron 9 spliced before intron 7 also has 18 reads in support (Fig. 3a). Although splicing is a relatively out of order process, there is clearly a preferred order, e.g., intron 1 spliced before intron 4 has 1 read in support, while intron 4 spliced before intron 1 has 12 reads in support. This result is consistent with a recent study that analysing the intron splicing order pairs [8]. In addition, introns that are close to each other have more reads support than introns between long-range sequences (Fig. 3a). This is because the distances between intron pairs that are far from each other are usually very long and make sequencing of the whole region very difficult. The most likely order for *hnRNP A1* (ENST00000546500) is 4 → 3 → 8 → 5 → 1 → 6 → 9 → 7 → 2 with a Spearman Rho = − 0.16. Intron 2 is spliced last as predicted, and this is consistent with row 2 having relatively smaller values than column 2 (Fig. 3a). Another way to validate the most likely order is by ordering the frequency matrix and graph by the most likely order and then checking if the read counts are consistent with the order (Fig. 3b and Additional file 1). To quantitively measure the consistency between the calculated intron splicing order and the direction of transcription, the relative likelihood was calculated. The log relative likelihood of − 28 suggests that this transcript of *hnRNP A1* has little probability of being spliced in an order that is consistent with its transcriptional direction.

The full result of the most likely order can be checked in Additional file 2. These transcripts were selected because their intron splicing order pairs were well detected (the percentage of intron splicing order pairs retained > 95%) and only expressed isoforms are used in humans, *Arabidopsis thaliana* and fruit fly. The results showed that intron splicing orders change from one gene to another and are not consistent with the order of transcription (Additional file 2).

**Fig. 4:**
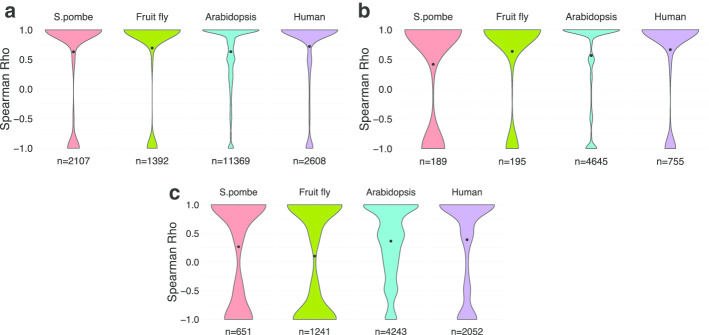
The stable of the detected intron splicing orders. **a** The dataset was randomly assigned into two groups, and then the intron splicing orders between the two groups were correlated to test whether the intron splicing order is stable between different RNA-seq datasets. **b** Correlation of the intron splicing orders between short-read sequencing and long-read sequencing. **c** Correlation between FPKM values of introns and the calculated intron spicing orders. The mean value is labeled as a black dot in each violin plot

One critical question is whether the intron splicing order is stable between different datasets. To test this, the whole dataset was randomly assigned into two groups to test the stability of the intron splicing order. The random assignment was repeated 5 times. The results showed that the intron splicing order is a stable process between different biological replicates (Fig. 4a). In addition, short-read sequencing was used here to confirm that the intron splicing order is robust with different techniques (Fig. 4b). Slower spliced introns have higher FPKM values than faster introns in total RNA-seq [2]. I correlated the FPKM of each intron with the calculated intron splicing orders in four organisms, the result showed an obvious positive correlation (Fig. 4c).

### Humans harbour more not in-order spliced transcripts than *S. pombe*, fruit fly, and *Arabidopsis thaliana*

**Fig. 5:**
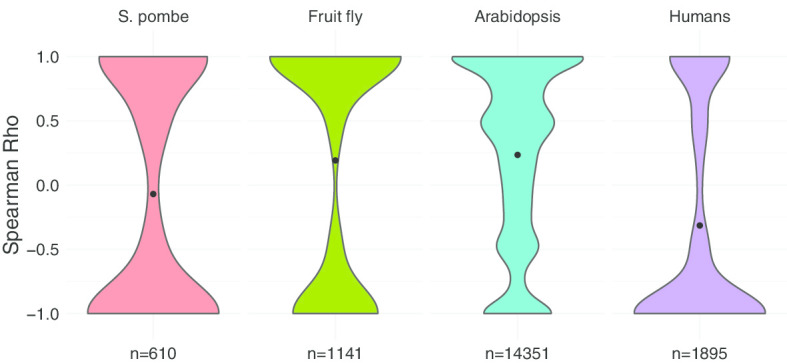
Human contains more not in-order spliced transcripts. Distribution of Spearman Rho in *S. pombe*, fruit fly, *Arabidopsis thaliana*, and humans. Splicing orders in humans tend to show a negative correlation with transcription direction

To systematically evaluate the in-order splicing properties of *S. pombe*, fruit fly, *Arabidopsis thaliana*, and humans, Spearman correlations were performed to further test this. The results showed that humans exhibit more negative Rho values than *S. pombe*, fruit fly, and *Arabidopsis thaliana*, which indicates that humans harbour more not in-order spliced transcripts (Fig. 5). Fruit fly and *Arabidopsis thaliana* harbour more in-order spliced transcripts while *S. pombe* has almost the same number of in-order spliced and not in-order spliced transcripts (Fig. 5). The trend was more obvious when doing this analysis for each RNA-seq separately (Additional file 1). One recent study also indicated that introns in fruit flies prefer to be co-transcriptional spliced than introns in humans and human introns tend to be spliced in a reverse direction [8].

### The first introns tend to be spliced slower in humans compared with the other three organisms

**Fig. 6:**
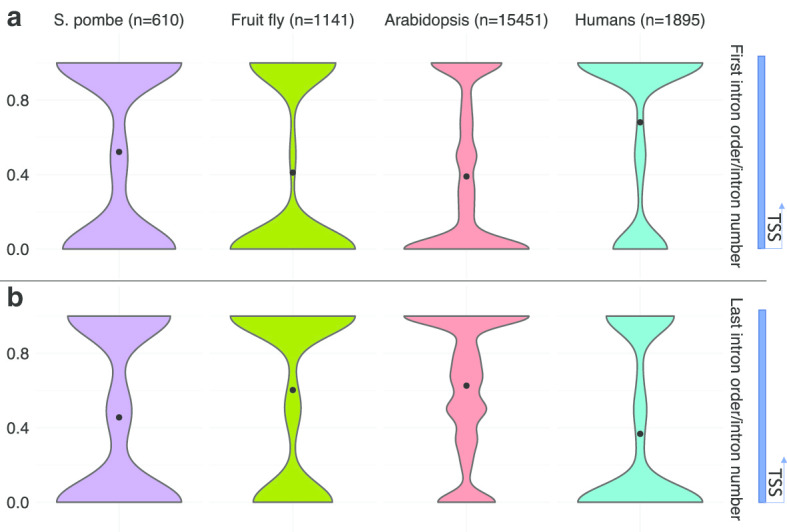
The first introns in humans tend to be spliced the slowest. **a** Distribution of the first introns’ relative splicing orders in *S. pombe*, fruit fly, *Arabidopsis thaliana*, and humans. **b** Distribution of the last introns’ relative splicing orders in *S. pombe*, fruit fly, *Arabidopsis thaliana*, and humans

The first intron plays a more important role in gene regulation than other introns in humans [13], and there are studies indicating that the first intron is usually spliced the slowest [6, 7]. To test this systematically, the relative orders were calculated by dividing the order of the first intron by the total intron number for each transcript and constructed violin plots (Fig. 6a). The results showed that the first intron tends to be spliced slower in humans than in the other organisms. The first introns in fruit flies and *Arabidopsis thaliana* tend to be spliced the fastest, while the first introns in *S. pombe* neither tend to be spliced the fastest nor the slowest (Fig. 6a). This result is consistent with the above result that human introns are spliced in a more not in-order manner than introns in the other organisms. The same relative orders were calculated for the last introns as a comparison (Fig. 6b). The trend was also more obvious when doing this analysis for each RNA-seq separately (Additional file 1).

**Fig. 7:**
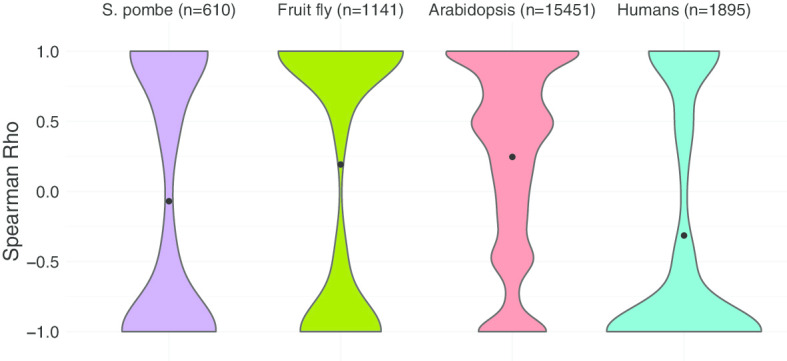
Correlation of the most likely orders with introns’ distance to TSS in *S. pombe*, fruit fly, *Arabidopsis thaliana*, and humans respectively

To determine the driving force behind the most likely intron splicing order, correlation analysis was performed between the calculated most likely orders with intron length, distances of the introns from the TSS, 5′ splice site scores, 3′ splice site scores, GC content in intron, upstream exon length, and downstream exon length. The distance to TSS in humans showed an apparently negative correlation with the most likely order, while this metric showed positive correlations with the most likely order in *Arabidopsis thaliana* and fruit fly (Fig. 7). This is consistent with the above result that intron splicing orders in humans are more not in-order than that in other organisms analysed here, and also agree with that the first introns tend to be spliced the fastest in *Arabidopsis thaliana* and fruit fly. The intron splicing orders in *S. pombe* didn’t correlate with this metric. Other metrics didn’t show an obvious correlation with the intron splicing orders (Additional file 1).

### Heterogeneity of intron splicing order in different organisms

**Fig. 8:**
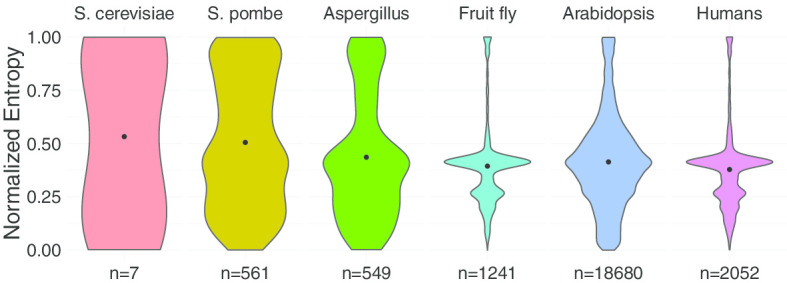
The heterogeneity of the intron splicing orders

The above results support that introns in fruit flies, *Arabidopsis thaliana* tend to be in-order spliced, while introns in humans tend to be spliced in a reverse direction. The introns in *S. pombe* didn’t prefer to be in-order or not in-order spliced. This suggests that intron splicing in *S. pombe* may not similar to other organisms analyzed here. A measure was calculated to obtain the heterogeneity of the intron splicing orders in each transcript for the four organisms. The results showed that the intron splicing orders in *S. pombe* are more heterogeneous, i.e., tends not to be spliced in a defined order compared with the other organisms (Fig. 8). The result is consistent with the above results on that *S. pombe* neither enriches in-order spliced nor not in-order spliced transcripts. The difference of the heterogeneity between *S. pombe* and the other genomes is not significant when redid this analysis for each RNA-seq (Additional file 1). So the heterogeneity of the intron splicing orders in *S. cerevisiae* and Aspergillus nidulans were also analysed here. The result showed that lower organisms tend to have higher entropy (Fig. 8).

### Webserver

I integrated this method into a web viewer to visualize intron splicing orders (Additional file 1). In this viewer, researchers can adjust the read count threshold as well as other parameters to adjust the most likely order calculation process. Researchers can also check the raw read count of the intron splicing order matrix and transcriptional structure of this gene in this viewer. The website address is [12].

## Discussion

In this work, a method that accurately calculate the intron splicing orders genome widely was developed. I found introns in humans are more not in-order spliced than the other three organisms, and the first introns in humans also tend to be spliced the slowest.

Higher organisms always contain a very high number of alternative splicing events, i.e., SE, A3SS, A5SS, MXE and RI, and these events make accurate calculation of intron splicing orders very difficult, but this bias will be reduced as sequencing reads become longer. Current long-read sequencing technology or short-read mate-pair sequencing (short-read sequencing can only sequence the two ends of a fragment) can sequence fragments over 10,000 bp [14], and these fragments can contain millions of nucleotides if we avoid the constraints of reverse transcriptase by direct RNA sequencing. Short-read are always cheaper and the throughput is much higher, but long-read can detect more introns and more accurately for the intron splicing order problem. The fragment size of paired-end sequencing in the Illumina sequencer can be very long in theory, but the accuracy decreases dramatically when fragment size > 500 bp [15]. In addition, sequencing experiments are always performed for a specific tissue or cell line each time, and each cell line only expresses a small part of the transcripts, which will further reduce the bias. Another factor is that we cannot distinguish most isoforms of the same gene until alternative splicing occurs. Thus, in higher organisms, transcripts that share many introns and are expressed in the same tissue/cell line have to be treated as having similar intron splicing orders based on current techniques.

This study is limited because only a small part of the transcriptome is covered here in fruit fly and humans, and the transcripts analysed here usually contain short and rare introns compared with the whole transcriptome. This may lead to some bias.

## Conclusions

This is the first method that can calculate intron splicing orders. The method requires a batch of pre-mRNA long-read sequencing datasets to detect intron splicing orders. One of the major applications will be the use of this method on a few interesting genes to determine the relationships between intron splicing orders, splicing fidelity, secondary structure, and alternative splicing. By applying the method on real sequencing datasets, intron splicing orders were calculated for four different organisms. I found that the splicing orders in humans are more not in-order than those in the other three organisms. This is further supported by the fact that the first introns in humans tend to spliced the slowest. I found the intron splicing orders in *S. pombe* are different with the other organisms, further analysis showed lower organisms have more introns that are spliced not in a defined order.

## Methods

First, a method was proposed based on computation theory that can calculate intron splicing orders. Then, this method was applied to real sequencing datasets to calculate intron splicing orders for four organisms. The overall computation workflow is shown in Fig. 1 and Additional file 1. I list the detailed methods of each step in the following sections.

### Intron splicing order pair detection algorithm

A custom JAVA script was written that can calculate each pair of intron splicing orders in every transcript. The detailed intron splicing order pair detection algorithm was listed in Additional file 2. Steps 3–6 detect reads that support that intron j is not spliced, and steps 7–9 determine whether the above reads support that other introns are already spliced (contain junctions). This algorithm is used for both short-read paired-end sequencing and long-read sequencing datasets.

### Intron splicing order graph

The intron splicing order graph is based on the R packages igraph, ggraph and networkD3.

### Most likely order calculation model and algorithm

As described above, the read counts are not uniformly distributed in the intron splicing order matrix. The read counts decrease as they move away from the main diagonal, and the decay rate is different from gene to gene due to different intron and exon lengths, which makes modelling the decay rate very difficult. An additional consideration is that the read coverage should not influence the calculated most likely order. Thus, a frequency-based method was used here to calculate the most likely intron splicing orders. The idea is that for each type of order of introns, a probability can be calculated, and the order with the highest probability is the most likely order. For example, if the order is 1 → 3 → 2, then this also implies that intron 1 is spliced before 2, so a total of three intron splicing order pairs will be calculated. Let n = number of introns. For a specific order, the total number of intron splicing order pairs that need to be multiplied is $\frac{{n \times \left( {n - 1} \right)}}{2}$. Let $i \in 1,2,3, \ldots ,n$ and $j \in 1,2,3, \ldots ,n$ be the indices of introns. Let A be the read count adjacent matrix and D be the frequency matrix, where $D_{ij} = A_{ij} /\left( {A_{ij} + A_{ji} } \right)$ and $D_{ij} = 0$ when i = j. Another way to obtain D is by calculating the MLE by assuming a binomial distribution, i.e., $$D_{i,j} = \mathop {{\text{argmax}}}\limits_{\psi } \left( {\begin{array}{*{20}c} {A_{i,j} + A_{j,i} } \\ {A_{i,j} } \\ \end{array} } \right)\psi^{{A_{i,j} }} \left( {1 - \psi } \right)^{{A_{j,i} }}$$. Let $\theta = O_{1} ,O_{2} , \ldots ,O_{n}$ be an order of introns. There will be $n!$ different orders of introns for this transcript. The model is a kind of simplified Bayesian network, and the likelihood values between different types of orders can be treated as the likelihood values between different DAGs in the Bayesian network (Additional file 1). For a specific order $\theta = O_{1} ,O_{2} , \ldots ,O_{n}$, the probability can be calculated as follows. If $D_{ij} + D_{ji} \ne 0$, then either there is no read for the intron pair (intron i and intron j) or i = j.

1 $$P\left( {i\,spliced\, before\, j} \right) = P\left( {O_{i} < O_{j} } \right) = D_{ij}$$

2 $$\begin{aligned} {\text{L}}(\theta {|}D{)} & = P\left( {D{|}\theta } \right) \propto P\left( {\theta {|}D} \right) \propto \mathop \prod \limits_{i = 1}^{n} \mathop \prod \limits_{j = 1}^{n} P((O_{i} < O_{j} )|D) \\ & = \quad \mathop \prod \limits_{i = 1}^{n} \mathop \prod \limits_{j = 1}^{n} ( D_{ij} I[O_{i} < O_{j} ;D_{ij} + D_{ji} \ne 0] + I[O_{i} > O_{j} ;D_{ij} + D_{ji} \ne 0] \\ & \quad \quad + I[D_{ij} + D_{ji} = 0] )\quad {\text{where}}\,\,P\left( \theta \right) = \frac{1}{n!} \\ \end{aligned}$$

This optimization problem can be converted into $\theta^{*} = \mathop {{\text{argmax}}}\limits_{permutation} \left( { sum \left( {upper\,triangle\left( {\log D} \right)} \right)} \right)$, where permutation refers to the swapping of row i to row j and column i to column j simultaneously. The most unlikely order is listed below. The most unlikely order is the reverse of the most likely order.

3 $$\begin{aligned} \hat{\theta } & = \mathop {{\text{argmin}}}\limits_{permutation} \left( { sum \left( {upper\,triangle\left( {\log D} \right)} \right)} \right) \\ & = \mathop {{\text{argmin}}}\limits_{permutation} \left( { sum\left( {\log D} \right) - sum \left( {lower\,triangle\left( {\log D} \right)} \right)} \right) \\ & = sum\left( {\log D} \right) - \mathop {{\text{argmax}}}\limits_{permutation} \left( { sum \left( {lower\,triangle\left( {\log D} \right)} \right)} \right) \\ \end{aligned}$$

The optimization problem proposed here is very similar to the linear tournament ordering problem (Kemeny–Young problem) and the Bayesian network structure optimization problem, both are NP-hard problems [16, 17]. Permutation and calculation of all the possible intron splicing orders are needed, selecting the ones with the highest probability, which will be difficult for transcripts that contain intron numbers larger than 11. I used two algorithms to overcome this problem: for transcripts that contained intron number < 12, a permutation of all types of orders was used ($O\left( {n!} \right))$; for transcripts that contained 12 $\le$ intron number < 100, an integer linear programming approach was used, and over 99,97% of the transcripts in humans contained intron number < 100. The detailed formulation of the integer linear programming used here is the same as the one described in [18].

Another problem of this model is that some intron splicing order pairs have zero values. In this case, I added a small number (adjust_value = 0.1) on both sides for each intron pair, i.e.$,{ }D_{ij} = (A_{ij} + 0.1)/\left( {A_{ij} + A_{ji} + 2 \times 0.1} \right)$. Adjust_value = 0.1 can also be treated as a prior to the read count matrix, i.e.,. $$D_{i,j} = \mathop {{\text{argmax}}}\limits_{\psi } \left( {\begin{array}{*{20}c} {A_{i,j} + A_{j,i} } \\ {A_{i,j} } \\ \end{array} } \right)\psi^{{A_{i,j} }} \left( {1 - \psi } \right)^{{A_{j,i} }} beta\left( {\psi ,\alpha = 1.1,\beta = 1.1} \right)$$, and $\alpha$ and $\beta$ can be treated as pseudo read counts. Thus, the estimation of D can be treated as a calculation of the MAP of the binomial model with known beta priors.

To measure the in-order splicing probability, the relative likelihood values were calculated. Let $\hat{\theta } = O_{1} < O_{2} \cdots < O_{n}$ be the order of in-order splicing, let $\theta$ be an arbitrary order. If $\theta^{*}$ is the calculated optimum order, the log relative likelihood is listed as follows:

4 $$R\left( \theta \right) = {\ln}\left( {\frac{{L\left( {O_{1} < O_{2} \cdots < O_{n} {|}D} \right)}}{{L\left( {\theta^{*} {|}D} \right)}}} \right)$$

### Simulation study for *S. pombe* and human chr21

For each multi-intron containing transcript in *S. pombe*, first, the sequences of all the potential pre-mRNAs given a random defined order were obtained, e.g., if a transcript has 4 introns, then there will be 5 possible pre-mRNAs. Then, the reads from these pre-mRNAs were simulated using the R package polyester [19]. Please check Additional file 1 for the overall simulation process, 150-bp paired-end reads and fragment size of 800 was used for short-read sequencing, read length of 800 and fragment size of 6000 was used for long-read sequencing, and read length of 5000 and fragment size of 6000 was used for super long-read sequencing. To save on computational effort, only reads from ~ 1300 multi-introns containing transcripts were simulated in *S. pombe* and ignored transcripts that contained no introns or only one intron.

The chr21 in the human genome was chosen to do simulation because it is the shortest primary scaffold in the human genome except for chrY. I selected one transcript per gene for simulation and considered only transcripts that contained 2 or more introns. A read length of 150 bp and fragment size of 15,000 was used for short-read mate-pair, read length of 800 and fragment size of 15,000 was used for long-read sequencing, and read length of 12,000 and fragment size of 15,000 was used for super long-read sequencing.

To evaluate the situation in which there are some undetected intron splicing order pairs in the intron splicing order read count matrix in the real dataset, the read count matrixes were simulated directly using the Poisson distribution (mean of 15 reads per intron splicing order pair) given a random defined order. The simulation was performed in a total of 1000 times, with an average intron number of 7.

### Datasets and pre-processing for *S. pombe*, fruit fly, *Arabidopsis thaliana* and humans

As the above simulation results showed, paired-end sequencing is sufficient for calculating intron splicing orders in *S. pombe*; thus, both short-read and long-read total RNA-seq (SRP093735, SRP062858 and GSE104681) were used to calculate the intron splicing orders in *S. pombe*. The long-read sequencing reads (PRJNA591665) were used for *Arabidopsis thaliana*. The long-read nascent RNA sequencing reads (GSE123191) were used for fruit fly and humans. ENSEMBL Fungi annotation ASM29v2.43 [20] was used for *S. pombe*; ENSEMBL dm6 annotation was used for fruit fly; ENSEMBL TAIR10 annotation was used for *Arabidopsis thaliana*; and GENCODE annotation (hg19) was used for humans. STAR [21] was used for short-read alignment. The long-read sequencing reads were aligned using minimap2 [22] with parameters -ax splice -uf -k14. The BAM files were sorted and indexed by samtools [23]. The list of the full datasets used here can be found in Additional file 2.

### The detailed method for correcting the bias of retained introns

For humans, *Arabidopsis thaliana*, and fruit flies, many alternative splicing events exist in these organisms, and transcripts were filtered by TPM > 0.1. The TPM values are calculated using the TPMcalculator. The datasets came from the ENCODE and NCBI databases (human: ENCSR000AEO, fruit fly: ENCSR045CJI, *Arabidopsis thaliana*: SRR10538404). Among the different kinds of alternative splicing events, intron retention has a stronger effect on intron splicing order. Three conditions must be met to enable read that mapped into intron retention regions leading to bias in intron splicing order: (1). Both alternative isoforms are expressed in this dataset. (2). The two alternative isoforms share some common introns (check Additional file 1 for example). (3). The read supports retained introns spliced after other introns. To reduce the bias caused by retained introns, PSI values of the retained introns were calculated from polyA mRNA-seq. The PSI values can be treated as the percentile of reads assigned to the retained intron isoforms. This value is then used to correct the read count that supports the retained intron spliced after other introns. For example, the number of reads supporting the retained intron spliced after the other intron is ${\max}\left( {b - PSI \times \left( {b + c + d} \right),0} \right)$, where b represents read count detected as retained intron was first spliced; c represents read count detected as retained intron was slower spliced; d represents read count detected as both introns were spliced (Additional file 1). The intron retention events were extracted from annotations using rMATS [24].

### The intron splicing order calculation method developed here is error tolerance

Even if several pairs of intron splicing orders were wrong, the overall intron splicing order can be corrected. This is because the overall information in the intron splicing order pairs matrix is redundant, and not every pair of intron spicing orders is needed to calculate the final intron splicing orders. For example, suppose that the correct intron splicing order is 1 → 2 → 3 → 4 and every intron splicing order pair has read support. Even if the read count between intron 1 and 4 is wrong, i.e., 4 → 1, the final result would still be 1 → 2 → 3 → 4 because the order of intron 1 and intron 4 is fixed by intron 2 and intron 3 (see Additional file 1 for another example).

### The stable of intron splicing order

All the datasets used here contains more than one biological replicates, for each organism the bam files were randomly assigned into two groups and redid the intron splicing order calculation to test if the intron splicing order is stable. The permutation was repeated 5 times. The short-read total RNA-seq datasets used here are available in Additional file 2. The FPKM values of introns were calculated using TPMcalculator.

### Correlation analysis and the relative orders of first introns

All the intron splicing order pairs detected were used for *S. pombe*, fruit fly, *Arabidopsis thaliana*, and humans. Some transcripts contain more than one type of most likely order that has the same highest likelihood value. Thus, only transcripts that contain unique highest likelihood values are kept for downstream real data analysis except the analysis of heterogeneity. This filter was applied with transcripts that contain intron numbers < 12. All the correlations used here are Spearman correlations. Relative order = (order with intron − 1)/(total intron number − 1).

### The heterogeneity measure of intron splicing order

The heterogeneity measure is based on the entropy of the likelihood values. For an order ${\theta }$, the likelihood is $L\left( {{\theta }{|}D} \right)$. The normalized likelihood and entropy is listed as follows. The heterogeneity measure can only be calculated for transcripts that contain intron number < 12, and this threshold covers most of the transcripts detected here. The dataset used for this analysis came from a single study for each organism to reduce the variance using different experiments.

5 $$\begin{aligned} & p\left( {{\theta }{|}D} \right) = \frac{{L\left( {{\theta }{|}D} \right)}}{{\mathop \sum \nolimits_{all \theta } L(\theta |D)}}).{ }\quad Entropy = \mathop \sum \limits_{all \theta } P\left( {{\theta }{|}D} \right) \times log_{2}^{{P\left( {{\theta }{|}D} \right)}} \\ & Normalized\,Entropy = \frac{Entropy}{{log_{2}^{n!} }}. n \,is\, number\, of\, introns \\ \end{aligned}$$

#### Webserver

The webpage is based on the shiny package in R. The transcript structures are based on the Sushi package [25]. The graph in the webpage is based on the R packages networkD3, igraph and ggraph.

## Supplementary information

**Additional file 1**: Supplementary figures: S1 How to detect intron splicing order pairs, S2 The workflow of the method, S3 Further simulation result, S4 Intron splicing order of *hnRNP A1*, S5 Supplementary figures related to Fig 5, S6 Supplementary figures related to Fig 6, S7 Supplementary figures related to Fig 7, S8 Supplementary figures related to Fig 8, S9 Website snapshot, S10 An example of how to calculate likelihood value, S11 A diagram of how to simulate reads, S12 How to reduce the bias of A3SS and A5SS, S13 A diagram of how retained introns lead to bias, S14 How to correct retained introns, S15 The robust of the method.

**Additional file 2**: Supplementary tables: S1 Intron splicing orders for S. *cerevisiae*, S2 Intron splicing orders for S. *pombe*, S3 Intron splicing orders for Aspergillus nidulans, S4 Intron splicing orders for fruit fly, S5 Intron splicing orders for *Arabidopsis* *thaliana*, S6 Intron splicing orders for humans, S7 The algorithm used to detect intron splicing order pairs, S8 The datasets used here.

## Data Availability

The source code of the method developed here has been deposited at https://github.com/limeng12/intron_order. The intron splicing orders calculated here can be checked at https://intron-splicing-order.online:3838/iso/.

## Abbreviations

A3SS: Alternative 3′ splicing event
A5SS: Alternative 5′ splicing event
DAG: Directed acyclic graph
FPKM: Fragments per kilobase per million
MAP: Maximum a posteriori estimation
PSI: Percent spliced in
RI: Retained intron event
SE: Skip exon event
TPM: Transcripts per million
TSS: Transcription start site
