# Supplementary material for: Calculating the most likely intron splicing orders in S. pombe, fruit fly, Arabidopsis thaliana, and humans
Source: BMC Bioinformatics. 2020 Oct 24;21:478. doi: 10.1186/s12859-020-03818-6 (PMC7585206; doi:10.1186/s12859-020-03818-6)
Supplement: Supplementary file 1 — Additional file 1: Supplementary figures: S1 How to detect intron splicing order pairs, S2 The workflow of the method, S3 Further simulation result, S4 Intron splicing order of hnRNP A1, S5 Supplementary figures related to Fig 5, S6 Supplementary figures related to Fig 6, S7 Supplementary figures related to Fig 7, S8 Supplementary figures related to Fig 8, S9 Website snapshot, S10 An example of how to calculate likelihood value, S11 A diagram of how to simulate reads, S12 How to reduce the bias of A3SS and A5SS, S13 A diagram of how retained introns lead to bias, S14 How to correct retained introns, S15 The robust of the method. [file 12859_2020_3818_MOESM1_ESM.docx]

Supplementary Figure 1. A) Reads support that intron 1 is spliced before intron 3. B) Reads support that intron 3 is spliced before intron 1. A read must contain at least one junction to detect the splicing order of a pair of introns. The fragment size must at least be the size of an exon to detect intron splicing order pairs.

Supplementary Figure 2. The workflow of the method.

Supplementary Figure 3. Simulation result based on chr21 in humans. The number of reads for each transcript of each simulation is labelled at the bottom. Read length is more important than read count. The total number of reads for each simulation is $\#reads\times20K$.

Supplementary Figure 4. Intron splicing order of *hnRNP A1*. A) The intron splicing order read count graph of *hnRNP A1*. The intron index number is labelled in the vertex, and arrow from 1 to 2 means intron 1 spliced before intron 2. The width of the arrow line represents the read count supports this intron splicing order pair. B) Reordering the intron splicing order read count graph by the most likely order, arrows above the vertexes point to the slower-spliced introns, arrows below the vertexes point to faster-spliced introns. The values above the vertexes are larger than the values below the vertexes, this supports the most likely order calculated.

Supplementary Figure 5. Corresponding to Figure 5, each RNA-seq dataset was run separately to check whether the direction of splicing was consistent with the transcription direction. A) *S. pombe*. B) Fruit fly. C) *Arabidopsis thaliana*. D) Humans.

Supplementary Figure 6. Corresponding to Figure 6, each RNA-seq dataset was run separately to check the splicing order of the first introns. A) *S. pombe*. B) Fruit fly. C) *Arabidopsis thaliana*. D) Humans.

Supplementary Figure 7. Correlation of the calculated most likely orders with introns’ length, introns’ distance to TSS, 5’ splice site maxEntScore, 3’ splice site maxEntScore, intron GC content, upstream exon length and downstream exon length in S. *pombe*, fruit fly, *Arabidopsis* *thaliana* and humans respectively.

Supplementary Figure 8. Corresponding to Figure 8, the heterogeneity of the intron splicing orders for each RNA-seq dataset. A) S. *pombe*. B) Fruit fly. C) S. *cerevisiae*. D Aspergillus nidulans. E) Humans. F) *Arabidopsis* *thaliana*.


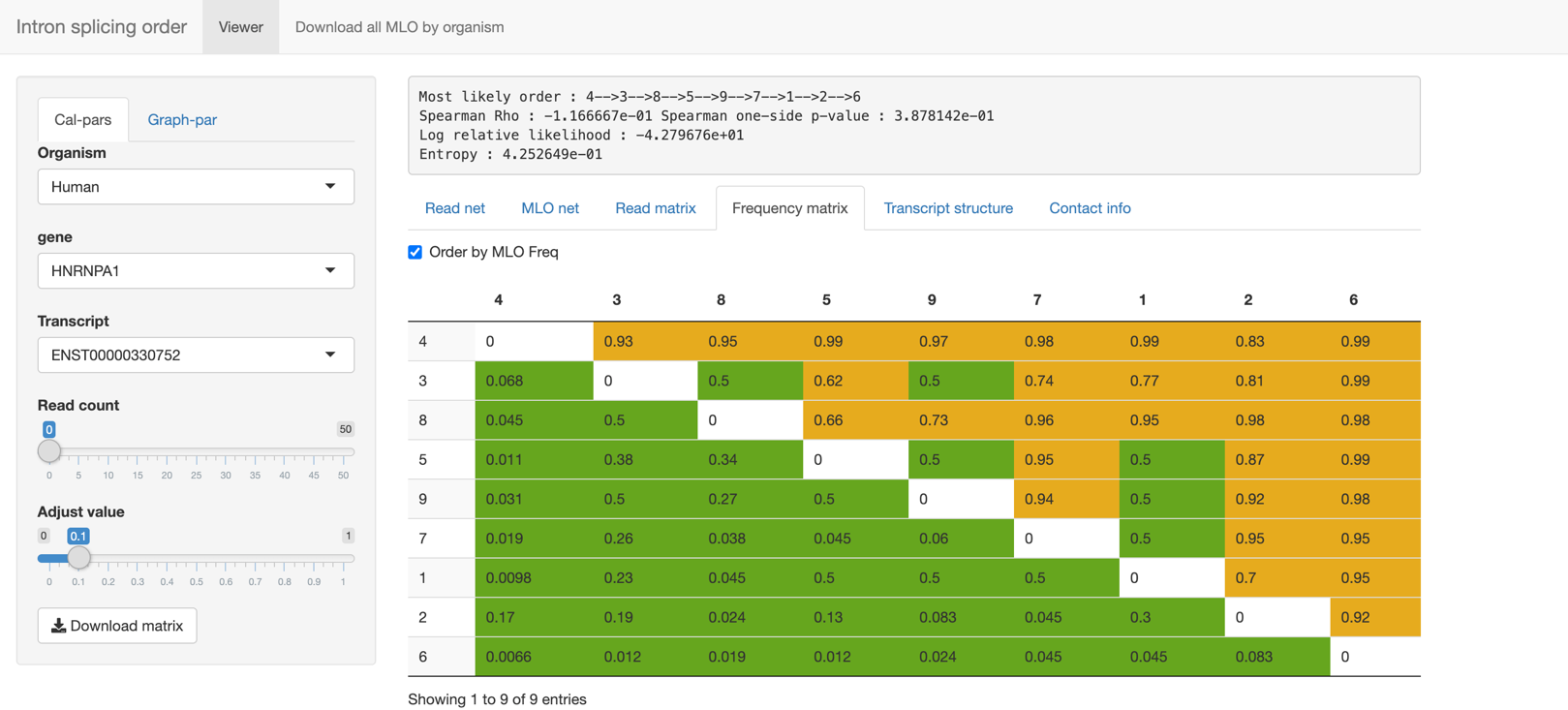


Supplementary Figure 9. Website snapshot.

Supplementary Figure 10. An example of the method used here to calculate the likelihood values for two type of orders.

Supplementary Figure 11. A diagram of the method used here to simulate fragments and reads from pre-mRNA given defined order.

Supplementary Figure 12. The method used to reduce the bias of A3SS, A5SS, and SE events. The idea is enforcing the reads that mapped into the exon-intron border region with enough length.

Supplementary Figure 13. Only some introns need to be adjusted for intron retention. Introns that are shared between the inclusion and exclusion isoform need to be corrected. In this figure, only intron 2 needs to adjust the read counts (corresponding to read 1), since read 2 read 3, and read 4 cannot belong to isoform 1.

Supplementary Figure 14. The method used here to correct retained introns, where a, b, c, and d are the read counts of the corresponding pre-mRNA. Arrows represent the process of splicing.

Supplementary Figure 15. The robustness of the method. The most likely order of the lower transcript (ENST00000546500) is 4→3→8→5→1→6→9→7→2. The bias of intron retention came from intron 2. The read count support intron splicing order of introns 2⇔1, 2⇔3 and 2⇔4 can be incorrect, but since the order of intron 2 is fixed by 8→5→1→6→9→7, regardless of the read count between introns 2⇔3 or introns 2⇔4, the overall order does not change. The read counts of introns 2⇔3 and 2⇔4 are highlighted in orange.
